# DeepBlueR: large-scale epigenomic analysis in R

**DOI:** 10.1093/bioinformatics/btx099

**Published:** 2017-02-22

**Authors:** Felipe Albrecht, Markus List, Christoph Bock, Thomas Lengauer

**Affiliations:** 1 Max Planck Institute for Informatics, Saarland Informatics Campus, Saarbrücken, Germany; 2 Graduate School of Computer Science, Saarland Informatics Campus, Saarbrücken, Germany; 3 CeMM Research Center for Molecular Medicine of the Austrian Academy of Sciences, Medical University of Vienna, Vienna, Austria; 4 Department of Laboratory Medicine, Medical University of Vienna, Vienna, Austria

## Abstract

**Motivation:**

While large amounts of epigenomic data are publicly available, their retrieval in a form suitable for downstream analysis is a bottleneck in current research. The DeepBlue Epigenomic Data Server provides a powerful interface and API for filtering, transforming, aggregating and downloading data from several epigenomic consortia.

**Results:**

To make public epigenomic data conveniently available for analysis in R, we developed an R/Bioconductor package that connects to the DeepBlue Epigenomic Data Server, enabling users to quickly gather and transform epigenomic data from selected experiments for analysis in the Bioconductor ecosystem.

**Availability and Implementation:**

http://deepblue.mpi-inf.mpg.de/R.

**Requirements:**

R 3.3, Bioconductor 3.4.

**Supplementary information:**

Supplementary data are available at *Bioinformatics* online.

## 1 Introduction

Epigenomic mapping consortia such as the BLUEPRINT Epigenome Project (Adams *et al.*, 2012), the German Epigenome Programme (DEEP) (http://www.deutsches-epigenom-programm.de), The Encyclopedia of DNA Elements (ENCODE) (The ENCODE Project Consortium, 2004) and the NIH Roadmap Epigenomics Mapping Consortium (ROADMAP) (Kundaje *et al.*, 2015) have made substantial progress in generating epigenomic data. These individual projects cooperate under the International Human Epigenome Consortium (IHEC) (Stunnenberg *et al.*, 2016) with the goal to define standards for data quality, metadata content and processing pipelines, as well as to make processed data available to the scientific community. For the latter, a number of data portals have been developed (Bujold *et al.*, 2016; Fernández *et al.*, 2016) through which relevant experimental data can be downloaded for local analyses. However, this approach has certain disadvantages. For instance, huge files that span the entire genome need to be downloaded even if only a small portion is needed, e.g. only promoter regions. Moreover, to answer a specific research question, it is usually necessary to transform, filter and aggregate data of various types across many experimental files. Complex operations on these data are not always feasible on a local computer due to resource limitations. To facilitate the analysis of public epigenomic datasets, we previously developed the DeepBlue epigenomic data server (Albrecht *et al.*, 2016), a platform that provides programmatic access to unaltered epigenomic data provided by the aforementioned consortia and to server-side data operations through a web service.

R (R Core Team, 2016) and the Bioconductor ecosystem (Huber *et al.*, 2015) form one of the most popular environments for downstream analysis and visualization of genomic and epigenomic data. Access to epigenomic data from various sources is already possible through the AnnotationHub package (http://bioconductor.org/packages/AnnotationHub/), for instance. However, a general solution for extracting only relevant subsets of information as it is possible with the DeepBlue server is currently missing. Here we present a R/Bioconductor package that provides user-friendly access to DeepBlue and streamlines the workflow from data retrieval to downstream analysis.

## 2 Overview

**Fig. 1.**
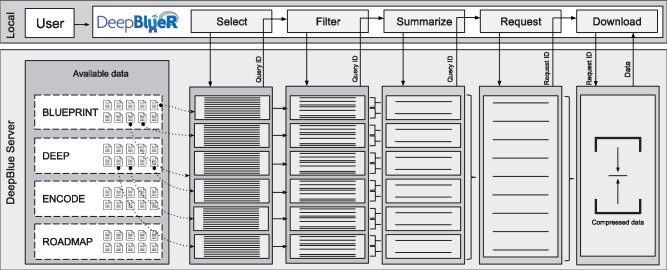
DeepBlueR facilitates combining data operations into a data processing workflow. For each command, a query ID is returned and the final data is accessible through the request ID

In DeepBlueR, various commands can be combined in custom workflows operating on epigenomic data on the DeepBlue server. A list of commands available DeepBlueR is provided in the Supplementary Information. DeepBlueR has been optimized for speed, which included modifications of the Bioconductor XML-RPC package, use of data compression and local caching of results. Upon import, all data is converted into suitable R data structures such as GenomicRanges (Lawrence *et al.*, 2013). In a typical workflow (Fig. 1), a set of regions is selected from various files. The selected regions are subsequently filtered and finally summarized. Each data operation command returns a *Query ID* that can either serve as input for the following command or can be used to trigger the execution of the workflow. In the latter case, a *Request ID* is returned which allows for checking if a request is completed and for downloading the results. DeepBlue incorporates commonly used annotations such as GENCODE (Harrow *et al.*, 2012) or the ENSEMBL regulatory build (Zerbino *et al.*, 2015) to simplify the selection of regions of interest.

## 3 Conclusion

Public data portals enable researchers to access to terabytes of epigenomic data. This creates a strong demand for data analysis in statistical environments such as R, which is not effective on local computers due to the volume of the data. Here we present a Bioconductor package that enables R users to tap directly into the DeepBlue epigenomic data server to operate on large epigenomic datasets. Results are conveniently transformed to R data structures that can be directly used with R/Bioconductor packages for visualization or analysis. Usage examples and documentation can be found in the Supplementary Information, including an example of a genome-wide cluster analysis of DNA methylation across 212 samples from the BLUEPRINT consortium. For the future, we intend to add new functionality as the DeepBlue API evolves. Moreover, we aim at providing better integration with R packages such as TCGAbiolinks (Colaprico *et al.*, 2015) or LOLA (Sheffield and Bock, 2016).

## Funding

This work has been supported by the German Federal Ministry of Education and Research grant no. 01KU1216A (DEEP project) and has been performed in the context of EU FP7 grant no. HEALTH-F5-2011-282510 (BLUEPRINT project).

*Conflict of Interest*: none declared.

## Supplementary Material

Supplementary Data
