# Supplementary material for: DeepBlueR: large-scale epigenomic analysis in R
Source: Bioinformatics. 2017 Feb 22;33(13):2063–4. doi: 10.1093/bioinformatics/btx099 (PMC5870546; doi:10.1093/bioinformatics/btx099)
Supplement: Supplementary Data [file btx099_supp.pdf]

DeepBlueR - DeepBlue Epigenomic Data Server - R package

Felipe Albrecht, Markus List

2017-03-09

Contents

Introduction **2**
What is DeepBlue ? 2

Getting started **2**
Installation 2
Overview of DeepBlue commands 3

DeepBlue usage examples **4**
Options 23
Caching 24

Large-scale analysis of DNA methylation across 212 samples from the BLUEPRINT consortium **24**
Aim 24
Dependencies 24
Select experiments 24
Select experiment column 25
Filter for genomic regions of interest using annotations 26
Generate a score matrix 27
Generating a heatmap 28

Further reading material **33**

Final remarks **33**

Figure 1:

Introduction

The DeepBlue Epigenomic Data Server is an online application that allows researchers to access data from various epigenomic mapping consortia such as DEEP, BLUEPRINT, ENCODE, or ROADMAP. DeepBlue can be accessed through a web interface or programmatically via its API. The usage of the API is documented with examples, use cases, and a user manual. While the description of the API is language agnostic, the examples and use cases shown online are focused on the python language. However, the R package presented here also enables access to the DeepBlue API directly within the R statistical environment and provides convenient functionality for triggering operations on the DeepBlue server as well as for data retrievel using R functions. In the following, we give a brief introduction to the package and subsequently show how python examples from the online documentation can be reproduced with it.

What is DeepBlue ?

A wealth of epigenomic data has been collected over the past decade by large epigenomic mapping consortia. Event though most of these data are publicly available, the task of identifiying, downloading and processing data from various experiments is challenging. Recognizing that these tedious steps need to be tackled programmatically, we developed the DeepBlue epigenomic data server. Epigenome data from the different epigenome mapping consortia are accessible with standardized metadata. An experiment is the most important entity in DeepBlue and typically encompasses a single file (usually a bed or wig file) with a set of mandatory metadata: name, genome assembly, epigenetic mark, biosource, sample, technique, and project. For the sake of organization, all metadata fields are part of controlled vocabularies, some of which are imported from ontologies (CL, EFO, and UBERON, to name a few). DeepBlue also contains annotations, i.e. auxiliary data that is helpful in epigenomic analysis, such as, for example, CpG Islands, promoter regions, and genes. DeepBlue provides different types of commands, such as listing and searching commands as well as commands for data retrieval. A typical work-flow for the latter is to select, filter, transform, and finally download the selected data. For a more thorough description of DeepBlue we refer to the DeepBlue publication in the 2016 NAR webserver issue. If you find DeepBlue useful and use it in your project consider citing this paper.

Important note: With the exception of data aggregation tasks, DeepBlue does not alter the imported data, i.e. it remains exactly as provided by the epigenome mapping consortia.

Getting started

Installation

Installation of DeepBlueR and its companion packages can be performed using the Bioconductor installer:

```
source("https://bioconductor.org/biocLite.R")
biocLite("DeepBlueR")
```

The package name is `DeepBlueR` and it can be loaded via:

```
library(DeepBlueR)
```

You can test your installation and connectivity by saying hello to the DeepBlue server:

```
deepblue_info("me")
```

Overview of DeepBlue commands

DeepBlue provides a comprehensive programmatic interface for finding, selecting, filtering, summarizing and downloading annotated genomic region sets. Downloaded region sets are stored using the GenomicRanges R package, which allows for downloaded region sets to be further processed, visualized and analyzed with existing R packages such as LOLA or GViz.

A list of all commands available by DeepBlue is provided in its API page. The vast majority of these commands is also available through this R package and can be listed as follows:

```
help(package="DeepBlueR")
```

In the following we listed the most frequently used DeepBlue commands. The full list of commands is available here. Note that each command in the following two tables has the prefix 'deepblue_*', e.g. deepblue_select_genes.

Category	Command	Description
Information	info	Information about an entity
List and search	list_genomes	List registered genomes
	list_biosources	List registered biosources
	list_samples	List registered samples
	list_epigenetic marks	List registered epigenetic marks
	list_experiments	List available experiments
	list_annotations	List available annotations
	search	Perform a full-text search
Selection	select_regions	Select regions from experiments
	select_experiments	Select regions from experiments
	select_annotations	Select regions from annotations
	select_genes	Select genes as regions
	select_expressions	Select expression data
	tiling_regions	Generate tiling regions
	input_regions	Upload and use a small region-set
Operation	aggregate	Aggregate and summarize regions
	filter_regions	Filter regions by theirs attributes
	flank	Generate flanking regions
	intersection	Filter for intersecting regions
	overlap	Filter for regions overlapping by at least a specific size
	merge_queries	Merge two regions set
Result	count_regions	Count selected regions
	score_matrix	Request a score matrix
	get_regions	Request the selected regions
	binning	Bin results according to counts
Request	get_request data	Obtain the requested data

In addition, this package provides a set of convenience functions not part of the DeepBlue API, such as:

Category	Command	Description
Request	batch_export_results	Download the results for a list of requests
	download_request_data	Download and convert the requested data (blocking)
	export_meta_data	Export metadata to a tab delimited file
	export_tab	Export any result as tab delimited file
	export_bed	Export GenomicRanges results as BED file

DeepBlue usage examples

In the following we give a number of increasingly complex examples illustrating what DeepBlue can achieve in your epigenomic data analysis work-flow. We go beyond the online description of these examples by showing how the retrieved information can be further used in R.

One of the first tasks in DeepBlue is finding the data of interest. This can be achieved in three ways:

- Using full-text search with the `deepblue_search` command
- Listing the available data with the `deepblue_list_{experiments, annotations, ...}` commands
- Using the companion DeepBlue web interface site for listing the data

Full-text search

In this example, we use the command `deepblue_search` to find experiments that contain the keywords 'H3k27AC', 'blood', and 'peaks' in their metadata. We put the names in single quotes to show that these names must be in the metadata.

```
# We are selecting the experiments with terms 'H3k27AC', 'blood', and
# 'peak' in the metadata.
experiments_found = deepblue_search(
    keyword="'H3k27AC' 'blood' 'peak'", type="experiments")

custom_table = do.call("rbind", apply(experiments_found, 1, function(experiment){
  experiment_id = experiment[1]
  # Obtain the information about the experiment_id
  info = deepblue_info(experiment_id)

  # Print the experiment name, project, biosource, and epigenetic mark.
  with(info, { data.frame(name = name, project = project,
    biosource = sample_info$biosource_name, epigenetic_mark = epigenetic_mark)
      })
}))
  head(custom_table)
```

```
##                          name             project biosource
## 1 E038-H3K27ac.narrowPeak.bed Roadmap Epigenomics     BLOOD
## 2 E047-H3K27ac.narrowPeak.bed Roadmap Epigenomics     BLOOD
## 3 E048-H3K27ac.narrowPeak.bed Roadmap Epigenomics     BLOOD
## 4 E037-H3K27ac.narrowPeak.bed Roadmap Epigenomics     BLOOD
## 5 E045-H3K27ac.narrowPeak.bed Roadmap Epigenomics     BLOOD
## 6 E040-H3K27ac.narrowPeak.bed Roadmap Epigenomics     BLOOD
##   epigenetic_mark
## 1         H3K27ac
## 2         H3K27ac
## 3         H3K27ac
## 4         H3K27ac
## 5         H3K27ac
## 6         H3K27ac
```

Listing experiments

We use the `deepblue_list_experiments` command to list all experiments with the corresponding values in their metadata.

```
experiments = deepblue_list_experiments(type="peaks", epigenetic_mark="H3K4me3",
    biosource=c("inflammatory macrophage", "macrophage"),
    project="BLUEPRINT Epigenome")
```

Accessing the extra-metadata

The extra-metadata is important because it contains information that is not stored in the mandatory metadata fields. We use the `deepblue_info` command to access an experiment's metadata- and extra-metadata fields. The following example prints the `file_url` attribute that is contained in the data imported from the ENCODE project.

```
info = deepblue_info("e30000")
print(info$extra_metadata$file_url)
```

```
## [1] "https://www.encodeproject.org/files/ENCFF001YBB/"
```

Select epigenomic data

We use the `deepblue_select_experiments` command to select all genomic regions from the two informed experiments. We use the `deepblue_count_regions` command with the `query_id` value returned by the `deepblue_select_experiments` command.

The `deepblue_count_regions` command is executed asynchronously. This means that the user receives a `request_id` and should check the status of this request. In contrast to the command `deepblue_get_request_data`, the DeepBlueR package-specific command `deepblue_download_request_data` will wait for the processing to finish, before downloading the data. Moreover, this command will convert any regions to a GRanges object.

```
query_id = deepblue_select_experiments(
    experiment_name=c("BL-2_c01.ERX297416.H3K27ac.bwa.GRCh38.20150527.bed",
        "S008SGH1.ERX406923.H3K27ac.bwa.GRCh38.20150728.bed"))
# Count how many regions where selected
request_id = deepblue_count_regions(query_id=query_id)
# Download the request data as soon as processing is finished
requested_data = deepblue_download_request_data(request_id=request_id)
print(paste("The selected experiments have", requested_data, "regions."))
```

```
## [1] "The selected experiments have 115347 regions."
```

Output with selected columns

We use the `deepblue_select_experiments` command to select genomic regions from the experiments that are in chromosome 1, position 0 to 50,000,000.

We then use the `deepblue_get_regions` command with the `query_id` value returned by the `deepblue_select_experiments` command to request the regions with the selected columns. Selecting the columns `@NAME` and `@BIOSOURCE` represent the experiment name and the experiment biosource.

The `deepblue_get_regions` command is executed asynchronously. This means that the user receives a `request_id` to be able to check for the status of this request. In contrast to the command `deepblue_get_request_data`, the DeepBlueR package-specific command `deepblue_download_request_data` will wait for the processing to finish, before downloading the data. Moreover, this command will convert any regions to a GRanges object.

```r
query_id = deepblue_select_experiments (
    experiment_name = c("BL-2_c01.ERX297416.H3K27ac.bwa.GRCh38.20150527.bed",
        "S008SGH1.ERX406923.H3K27ac.bwa.GRCh38.20150728.bed"),
        chromosome="chr1", start=0, end=50000000)

# Retrieve the experiments data (The @NAME meta-column is used to include the
# experiment name and @BIOSOURCE for experiment's biosource
request_id = deepblue_get_regions(query_id=query_id,
    output_format="CHROMOSOME,START,END,SIGNAL_VALUE,PEAK,@NAME,@BIOSOURCE")
regions = deepblue_download_request_data(request_id=request_id)
regions
```

```
## GRanges object with 3783 ranges and 4 metadata columns:
##          seqnames                 ranges strand | SIGNAL_VALUE      PEAK
##             <Rle>              <IRanges>  <Rle> |  <character> <integer>
##      [1]     chr1     [270668, 270987]      * |       6.5758        39
##      [2]     chr1     [271277, 271468]      * |       6.2148       136
##      [3]     chr1     [273768, 274209]      * |      14.1567       164
##      [4]     chr1     [778377, 778676]      * |       8.0198       154
##      [5]     chr1     [778409, 778678]      * |       4.5767       123
##      ...      ...                    ...    ... .          ...       ...
##   [3779]     chr1 [47437420, 47437621]      * |       3.7686       147
##   [3780]     chr1 [47437751, 47438038]      * |       9.6553       149
##   [3781]     chr1 [48245368, 48245867]      * |       4.7708       346
##   [3782]     chr1 [48542755, 48543280]      * |       7.3002       152
##   [3783]     chr1 [48793649, 48793986]      * |       5.1974       108
##                                                       @NAME   @BIOSOURCE
##                                                 <character>  <character>
##      [1] S008SGH1.ERX406923.H3K27ac.bwa.GRCh38.20150728.bed myeloid cell
##      [2] S008SGH1.ERX406923.H3K27ac.bwa.GRCh38.20150728.bed myeloid cell
##      [3] S008SGH1.ERX406923.H3K27ac.bwa.GRCh38.20150728.bed myeloid cell
##      [4] S008SGH1.ERX406923.H3K27ac.bwa.GRCh38.20150728.bed myeloid cell
##      [5] BL-2_c01.ERX297416.H3K27ac.bwa.GRCh38.20150527.bed         BL-2
##      ...                                                ...          ...
##   [3779] BL-2_c01.ERX297416.H3K27ac.bwa.GRCh38.20150527.bed         BL-2
##   [3780] S008SGH1.ERX406923.H3K27ac.bwa.GRCh38.20150728.bed myeloid cell
##   [3781] S008SGH1.ERX406923.H3K27ac.bwa.GRCh38.20150728.bed myeloid cell
##   [3782] S008SGH1.ERX406923.H3K27ac.bwa.GRCh38.20150728.bed myeloid cell
##   [3783] S008SGH1.ERX406923.H3K27ac.bwa.GRCh38.20150728.bed myeloid cell
##   -------
##   seqinfo: 1 sequence from an unspecified genome; no seqlengths
```

Filter epigenomic data by metadata

We use the `deepblue_list_samples` command to obtain all samples with the biosource 'myeloid cell' from the BLUEPRINT project. The `deepblue_list_samples` returns a list of samples with their IDs and content. We extract the sample IDs from this list and use it in the `deepblue_select_regions` command to selects genomic regions that are in chromosome 1, position 0 to 50,000.

Then, we use the `deepblue_get_regions` command with the parameter `query_id` returned by the `deepblue_select_regions` command and the columns `@NAME`, `SAMPLE_ID`, and `@BIOSOURCE` representing the experiment name, the sample ID, and the experiment biosource.

The `deepblue_get_regions` command is executed asynchronously. This means that the user re-

ceives a `request_id` to be able to check for the status of this request. In contrast to the command `deepblue_get_request_data`, the DeepBlueR package-specific command `deepblue_download_request_data` will wait for the processing to finish, before downloading the data. Moreover, this command will convert any regions to a GRanges object.

```
samples = deepblue_list_samples(
    biosource="myeloid cell",
    extra_metadata = list("source" = "BLUEPRINT Epigenome"))
samples_ids = deepblue_extract_ids(samples)
query_id = deepblue_select_regions(genome="GRCh38", sample=samples_ids,
    chromosome="chr1", start=0, end=50000)
request_id = deepblue_get_regions(query_id=query_id,
    output_format="CHROMOSOME,START,END,@NAME,@SAMPLE_ID,@BIOSOURCE")
regions = deepblue_download_request_data(request_id=request_id)
head(regions,1)
```

```
## GRanges object with 1 range and 3 metadata columns:
##       seqnames         ranges strand |
##          <Rle>      <IRanges>  <Rle> |
##   [1]     chr1 [10004, 10010]      * |
##                                                                  @NAME
##                                                            <character>
##   [1] S00D6311.plusStrandMulti.star_grape2_crg.GRCh38.20160531.bedgraph
##        @SAMPLE_ID   @BIOSOURCE
##       <character>  <character>
##   [1]      s10409 myeloid cell
##   -------
##   seqinfo: 1 sequence from an unspecified genome; no seqlengths
```

Filter epigenomic data by region attributes

We use the `deepblue_select_experiments` command for selecting genomic regions from two specific experiments that are in chromosome 1, position 0 to 50,000,000. Then, we filter these for regions with `SIGNAL_VALUE` > 10 and `PEAK` > 1000.

Then, we use the `deepblue_get_regions` command with the parameter `query_id` returned by the `deepblue_select_regions` command and the columns `@NAME` and `@BIOSOURCE` representing the experiment name and the experiment biosource.

The `deepblue_get_regions` command is executed asynchronously. This means that the user receives a `request_id` to be able to check for the status of this request. In contrast to the command `deepblue_get_request_data`, the DeepBlueR package-specific command `deepblue_download_request_data` will wait for the processing to finish, before downloading the data. Moreover, this command will convert any regions to a GRanges object.

```
query_id = deepblue_select_experiments(
    experiment_name = c("BL-2_c01.ERX297416.H3K27ac.bwa.GRCh38.20150527.bed",
        "S008SGH1.ERX406923.H3K27ac.bwa.GRCh38.20150728.bed"),
    chromosome="chr1", start=0, end=50000000)
query_id_filter_signal = deepblue_filter_regions(
    query_id=query_id, field="SIGNAL_VALUE", operation=">",
    value="10", type="number")
query_id_filters = deepblue_filter_regions(
    query_id=query_id_filter_signal, field="PEAK", operation=">",
    value="1000", type="number")
```

```r
request_id = deepblue_get_regions(query_id=query_id_filters,
    output_format="CHROMOSOME,START,END,SIGNAL_VALUE,PEAK,@NAME,@BIOSOURCE")
regions = deepblue_download_request_data(request_id=request_id)
regions
```

```
## GRanges object with 161 ranges and 4 metadata columns:
##         seqnames                 ranges strand | SIGNAL_VALUE      PEAK
##            <Rle>              <IRanges>  <Rle> |  <character> <integer>
##     [1]     chr1   [1142428, 1144001]      * |      10.9313      1275
##     [2]     chr1   [1573400, 1575582]      * |      17.8805      1094
##     [3]     chr1   [1612814, 1616174]      * |      32.2064      2802
##     [4]     chr1   [1668761, 1670450]      * |      20.2936      1017
##     [5]     chr1   [1778583, 1783797]      * |      35.4277      1293
##     ...      ...                    ...    ... .          ...       ...
##   [157]     chr1 [44774644, 44776655]      * |      16.3227      1160
##   [158]     chr1 [44806139, 44811000]      * |      22.8156      1381
##   [159]     chr1 [46301112, 46304262]      * |      19.8041      2397
##   [160]     chr1 [46579227, 46582046]      * |      15.9613      1824
##   [161]     chr1 [46593677, 46595181]      * |      11.8798      1304
##                                                   @NAME   @BIOSOURCE
##                                             <character>  <character>
##     [1] BL-2_c01.ERX297416.H3K27ac.bwa.GRCh38.20150527.bed         BL-2
##     [2] S008SGH1.ERX406923.H3K27ac.bwa.GRCh38.20150728.bed myeloid cell
##     [3] S008SGH1.ERX406923.H3K27ac.bwa.GRCh38.20150728.bed myeloid cell
##     [4] S008SGH1.ERX406923.H3K27ac.bwa.GRCh38.20150728.bed myeloid cell
##     [5] S008SGH1.ERX406923.H3K27ac.bwa.GRCh38.20150728.bed myeloid cell
##     ...                                                ...          ...
##   [157] S008SGH1.ERX406923.H3K27ac.bwa.GRCh38.20150728.bed myeloid cell
##   [158] S008SGH1.ERX406923.H3K27ac.bwa.GRCh38.20150728.bed myeloid cell
##   [159] S008SGH1.ERX406923.H3K27ac.bwa.GRCh38.20150728.bed myeloid cell
##   [160] S008SGH1.ERX406923.H3K27ac.bwa.GRCh38.20150728.bed myeloid cell
##   [161] S008SGH1.ERX406923.H3K27ac.bwa.GRCh38.20150728.bed myeloid cell
##   -------
##   seqinfo: 1 sequence from an unspecified genome; no seqlengths
```

Find intersecting regions

We use the `deepblue_select_experiments` command for selecting genomic regions from two specific experiments that are in chromosome 1, position 0 to 50,000,000. Then, we filter these for regions with `SIGNAL_VALUE` > 10 and `PEAK` > 1000.

The command `deepblue_intersection` filters for all regions of the `query_id` that intersect with at least one region in `promoters_id`.

Then, we use the `deepblue_get_regions` command with the parameter `query_id` returned by the `deepblue_select_regions` command and the columns `@NAME` and `@BIOSOURCE` representing the experiment name and the experiment biosource.

The `deepblue_get_regions` command is executed asynchronously. This means that the user receives a `request_id` to be able to check for the status of this request. In contrast to the command `deepblue_get_request_data`, the DeepBlueR package-specific command `deepblue_download_request_data` will wait for the processing to finish, before downloading the data. Moreover, this command will convert any regions to a GRanges object.

```r
query_id = deepblue_select_experiments(
    experiment_name = c("BL-2_c01.ERX297416.H3K27ac.bwa.GRCh38.20150527.bed",
        "S008SGH1.ERX406923.H3K27ac.bwa.GRCh38.20150728.bed"),
    chromosome="chr1", start=0, end=50000000)
promoters_id = deepblue_select_annotations(annotation_name="promoters",
    genome="GRCh38", chromosome="chr1")
intersect_id = deepblue_intersection(
    query_data_id=query_id, query_filter_id=promoters_id)
request_id = deepblue_get_regions(
    query_id=intersect_id,
    output_format="CHROMOSOME,START,END,SIGNAL_VALUE,PEAK,@NAME,@BIOSOURCE")
regions = deepblue_download_request_data(request_id=request_id)
regions
```

```
## GRanges object with 608 ranges and 4 metadata columns:
##         seqnames               ranges strand | SIGNAL_VALUE      PEAK
##            <Rle>            <IRanges>  <Rle> |  <character> <integer>
##     [1]     chr1     [903997, 904177]      * |       4.7708        89
##     [2]     chr1     [904302, 905111]      * |       5.4928       560
##     [3]     chr1     [910269, 910975]      * |       4.7201       136
##     [4]     chr1     [911973, 913915]      * |      17.0446       624
##     [5]     chr1     [923976, 924329]      * |       4.7201       109
##     ...      ...                  ...    ... .          ...       ...
##   [604]     chr1 [46718435, 46719027]      * |       6.2499       230
##   [605]     chr1 [47313340, 47313980]      * |       6.8711       174
##   [606]     chr1 [47313412, 47313588]      * |       4.8558       120
##   [607]     chr1 [47313632, 47314141]      * |      10.2801       371
##   [608]     chr1 [47333183, 47335172]      * |      18.9772       857
##                                                         @NAME   @BIOSOURCE
##                                                   <character>  <character>
##     [1] S008SGH1.ERX406923.H3K27ac.bwa.GRCh38.20150728.bed myeloid cell
##     [2] S008SGH1.ERX406923.H3K27ac.bwa.GRCh38.20150728.bed myeloid cell
##     [3] S008SGH1.ERX406923.H3K27ac.bwa.GRCh38.20150728.bed myeloid cell
##     [4] S008SGH1.ERX406923.H3K27ac.bwa.GRCh38.20150728.bed myeloid cell
##     [5] S008SGH1.ERX406923.H3K27ac.bwa.GRCh38.20150728.bed myeloid cell
##     ...                                                ...          ...
##   [604] BL-2_c01.ERX297416.H3K27ac.bwa.GRCh38.20150527.bed         BL-2
##   [605] BL-2_c01.ERX297416.H3K27ac.bwa.GRCh38.20150527.bed         BL-2
##   [606] S008SGH1.ERX406923.H3K27ac.bwa.GRCh38.20150728.bed myeloid cell
##   [607] S008SGH1.ERX406923.H3K27ac.bwa.GRCh38.20150728.bed myeloid cell
##   [608] S008SGH1.ERX406923.H3K27ac.bwa.GRCh38.20150728.bed myeloid cell
##   -------
##   seqinfo: 1 sequence from an unspecified genome; no seqlengths
```

```r
library(Gviz)
atrack <- AnnotationTrack(regions, name = "Intersecting regions",
    group = regions$`@BIOSOURCE`, genome="hg38")
gtrack <- GenomeAxisTrack()
itrack <- IdeogramTrack(genome = "hg38", chromosome = "chr1")
plotTracks(list(itrack, atrack, gtrack), groupAnnotation="group", fontsize=18,
           background.panel = "#FFFEDB", background.title = "darkblue")
```

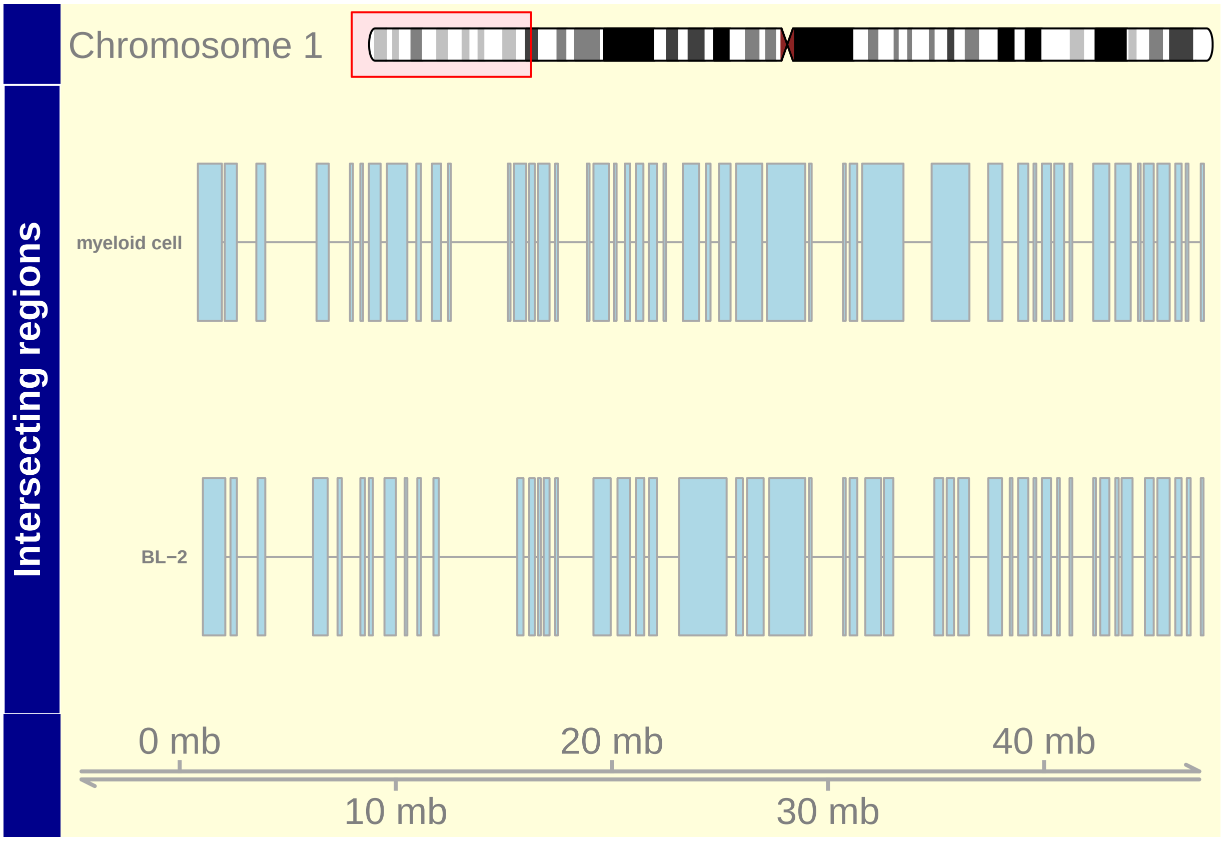

Retrieve DNA sequences

The meta-column `@LENGTH` contains the genomic region length, and we filter the genomic regions where this value is smaller than 2,000.

The meta-column `@SEQUENCE` includes the DNA Sequence in the genomic region output.

The `deepblue_get_regions` command is executed asynchronously. This means that the user receives a `request_id` to be able to check for the status of this request. In contrast to the command `deepblue_get_request_data`, the DeepBlueR package-specific command `deepblue_download_request_data` will wait for the processing to finish, before downloading the data. Moreover, this command will convert any regions to a GRanges object.

```
query_id = deepblue_select_experiments(
    experiment_name = c("BL-2_c01.ERX297416.H3K27ac.bwa.GRCh38.20150527.bed",
        "S008SGH1.ERX406923.H3K27ac.bwa.GRCh38.20150728.bed"),
    chromosome="chr1", start=0, end=50000000)
query_id_filter_signal = deepblue_filter_regions(query_id=query_id,
    field="SIGNAL_VALUE", operation=">", value="10", type="number")
query_id_filters = deepblue_filter_regions(query_id=query_id_filter_signal,
    field="PEAK", operation=">", value="1000", type="number")
query_id_filter_length = deepblue_filter_regions (query_id=query_id_filters,
    field="@LENGTH", operation="<", value="2000", type="number")
request_id = deepblue_get_regions(query_id=query_id_filter_length,
    output_format="CHROMOSOME,START,END,@NAME,@BIOSOURCE,@LENGTH,@SEQUENCE")
regions = deepblue_download_request_data(request_id=request_id)
head(regions, 1)
```

```
## GRanges object with 1 range and 4 metadata columns:
##       seqnames             ranges strand |
##          <Rle>          <IRanges>  <Rle> |
##   [1]     chr1 [1142428, 1144001]      * |
##                                                     @NAME  @BIOSOURCE
##                                               <character> <character>
##   [1] BL-2_c01.ERX297416.H3K27ac.bwa.GRCh38.20150527.bed        BL-2
##         @LENGTH
##       <integer>
##   [1]      1573
##
##
##   [1] CCAGGCTGGTCTCAAACTCCTGACCTCAAATGATCCGCCCACCTCGGCCTCCCACAGTGCTGGGATTACAGGCGTGAGTCACTGTGCCCGACCC
##   -------
##   seqinfo: 1 sequence from an unspecified genome; no seqlengths
```

DNA pattern matching operations

We use the `deepblue_find_pattern` command to find all position of a given pattern in the genome. An example is finding all locations of 'TATAA' in genome assembly GRCh38.

We use the `deepblue_select_experiments` command to select genomic regions that are in chromosome 1, position 0 to 50,000,000 from the selected experiments.

The command `deepblue_intersect` selects all regions of the `query_id` that intersect with at least one `tataa_regions` region.

```
tataa_regions = deepblue_select_annotations(
    annotation_name="Pattern TATAAA (non-overlap) in the genome GRCh38",
    genome="GRCh38", chromosome="chr1")
query_id = deepblue_select_experiments(
    experiment_name= c("BL-2_c01.ERX297416.H3K27ac.bwa.GRCh38.20150527.bed",
        "S008SGH1.ERX406923.H3K27ac.bwa.GRCh38.20150728.bed"),
    chromosome="chr1", start=0, end=50000000)
overlapped = deepblue_intersection(query_data_id=query_id,
                                   query_filter_id=tataa_regions)
request_id=deepblue_get_regions(overlapped,
    "CHROMOSOME,START,END,SIGNAL_VALUE,PEAK,@NAME,@BIOSOURCE,@LENGTH,@SEQUENCE,@PROJECT")
regions = deepblue_download_request_data(request_id=request_id)
head(regions, 3)
```

```
## GRanges object with 3 ranges and 7 metadata columns:
##       seqnames           ranges strand | SIGNAL_VALUE      PEAK
##          <Rle>        <IRanges>  <Rle> |  <character> <integer>
##   [1]     chr1 [273768, 274209]      * |      14.1567       164
##   [2]     chr1 [911203, 911477]      * |       4.8558       105
##   [3]     chr1 [916813, 917682]      * |      10.5467       183
##                                                     @NAME   @BIOSOURCE
##                                               <character>  <character>
##   [1] S008SGH1.ERX406923.H3K27ac.bwa.GRCh38.20150728.bed myeloid cell
##   [2] S008SGH1.ERX406923.H3K27ac.bwa.GRCh38.20150728.bed myeloid cell
##   [3] S008SGH1.ERX406923.H3K27ac.bwa.GRCh38.20150728.bed myeloid cell
##         @LENGTH
##       <integer>
##   [1]       441
```

```
##   [2]       274
##   [3]       869
##
##
##   [1]
##   [2]
##   [3] TTTATTGCACAAATTATTAACGACCAGAGAATGAATGACTCTGTAATCAGATCAGGTTGCCAGCACTTTTCATTGCATTTATTTGTATAAATTC
##                   @PROJECT
##                <character>
##   [1] BLUEPRINT Epigenome
##   [2] BLUEPRINT Epigenome
##   [3] BLUEPRINT Epigenome
##   -------
##   seqinfo: 1 sequence from an unspecified genome; no seqlengths
```

Counting motifs in a region

The meta column `@COUNT.MOTIF()` allows for counting how many times a motif appears in the selected genomic region. For example, the following code return the experiment regions with the DNA sequence length, the counts of `G`, `CG`, `GC`, and the DNA sequence itself.

```
experiment_data = deepblue_select_experiments(
  "DG-75_c01.ERX297417.H3K27ac.bwa.GRCh38.20150527.bed")
fmt = "CHROMOSOME,START,END,@LENGTH,@COUNT.MOTIF(C),@COUNT.MOTIF(G),@COUNT.MOTIF(CG),@COUNT.MOTIF(GC),@S
request_id=deepblue_get_regions(experiment_data, fmt)
regions = deepblue_download_request_data(request_id=request_id)
head(regions, 3)
```

```
## GRanges object with 3 ranges and 6 metadata columns:
##       seqnames           ranges strand |   @LENGTH @COUNT.MOTIF(C)
##          <Rle>        <IRanges>  <Rle> | <integer>     <character>
##   [1]     chr1 [779094, 779379]      * |       285              83
##   [2]     chr1 [826755, 827064]      * |       309             109
##   [3]     chr1 [958700, 959105]      * |       405             121
##       @COUNT.MOTIF(G) @COUNT.MOTIF(CG) @COUNT.MOTIF(GC)
##           <character>      <character>      <character>
##   [1]              85               15               24
##   [2]              78               15               18
##   [3]             124               29               32
##
##
##   [1]
##   [2]
##   [3] GAGATTTTTGCACAACTCACCAACATACGCTCCCTGCCTAGGACAGAGTTTGGCACGGAACAGGAGCTCAGTAAACATCGGATGAAAGAGTAAG
##   -------
##   seqinfo: 36 sequences from an unspecified genome; no seqlengths
```

Genes

We use the `deepblue_select_genes` command to select the gene `RP11-34P13` from GENCODE v23.

The selected genes behave like a regular genomic region, which, for example, can be filtered by their attributes. We use the `@GENE_ATTRIBUTE` meta-column to select the genomic regions that are annotated as lincRNAs.

```
q_genes = deepblue_select_genes(genes="RP11-34P13", gene_model="gencode v23")
q_filter = deepblue_filter_regions(query_id=q_genes,
    field="@GENE_ATTRIBUTE(gene_type)", operation="==",
    value="lincRNA", type="string")
request_id=deepblue_get_regions(q_filter, "CHROMOSOME,START,END,GTF_ATTRIBUTES")
regions = deepblue_download_request_data(request_id=request_id)
regions
```

Aggregate and summarize regions

The command deepblue_aggregate summarizes the query_id regions using the cpg_islands regions defined by the corresponding annotation as boundaries.

The aggregated values can be accessed through the @AGG.* meta-columns.

```
query_id = deepblue_select_experiments (
    experiment=c("GC_T14_10.CPG_methylation_calls.bs_call.GRCh38.20160531.wig"),
    chromosome="chr1", start=0, end=50000000)
cpg_islands = deepblue_select_annotations(annotation_name="CpG Islands",
    genome="GRCh38", chromosome="chr1", start=0, end=50000000)
# Aggregate
overlapped = deepblue_aggregate (data_id=query_id, ranges_id=cpg_islands,
    column="VALUE" )

# Retrieve the experiments data (The @NAME meta-column is used to include
# the experiment name and @BIOSOURCE for experiment's biosource
request_id = deepblue_get_regions(query_id=overlapped,
    output_format=
      "CHROMOSOME,START,END,@AGG.MIN,@AGG.MAX,@AGG.MEAN,@AGG.VAR")
regions = deepblue_download_request_data(request_id=request_id)
```

Gene expression

In the following example we obtain the gene expression levels of three genes, i.e., NOX3, NOXA1, and NOX4 from all biosources related to the hematopoietic stem cell biosource from the BLUEPRINT project. With related we refer to children of this biosource term in the ontologies used by DeepBlue.

```
hsc_children = deepblue_get_biosource_children("hematopoietic stem cell")

hsc_children_name = deepblue_extract_names(hsc_children)

hsc_children_samples = deepblue_list_samples(
    biosource = hsc_children_name,
    extra_metadata = list(source="BLUEPRINT Epigenome"))

hsc_samples_ids = deepblue_extract_ids(hsc_children_samples)

# Note that BLUEPRINT uses Ensembl Gene IDs
gene_exprs_query = deepblue_select_expressions(
    expression_type = "gene",
    sample_ids = hsc_samples_ids,
    identifiers = c("ENSG00000074771.3", "ENSG00000188747.7", "ENSG00000086991.11"),
    gene_model = "gencode v22")
```

```r
request_id = deepblue_get_regions(
    query_id = gene_exprs_query,
    output_format ="@GENE_NAME(gencode v22),CHROMOSOME,START,END,FPKM,@BIOSOURCE")

regions = deepblue_download_request_data(request_id = request_id)
regions
```

```
## GRanges object with 618 ranges and 3 metadata columns:
##         seqnames                 ranges strand | @GENE_NAME(gencode v22)
##            <Rle>              <IRanges>  <Rle> |             <character>
##     [1]     chr9 [137423350, 137434406]      * |                  NOXA1
##     [2]     chr9 [137423350, 137434406]      * |                  NOXA1
##     [3]     chr9 [137423350, 137434406]      * |                  NOXA1
##     [4]     chr9 [137423350, 137434406]      * |                  NOXA1
##     [5]     chr9 [137423350, 137434406]      * |                  NOXA1
##     ...      ...                    ...    ... .                    ...
##   [614]     chr6 [155395370, 155455903]      * |                   NOX3
##   [615]     chr6 [155395370, 155455903]      * |                   NOX3
##   [616]     chr6 [155395370, 155455903]      * |                   NOX3
##   [617]     chr6 [155395370, 155455903]      * |                   NOX3
##   [618]     chr6 [155395370, 155455903]      * |                   NOX3
##                FPKM                                   @BIOSOURCE
##         <character>                                  <character>
##     [1]      1.0700                                     monocyte
##     [2]      1.2300                     CD38-negative naive B cell
##     [3]      0.4600                   class switched memory B cell
##     [4]      0.6600                                memory B cell
##     [5]      2.1800 CD14-positive, CD16-negative classical monocyte
##     ...         ...                                          ...
##   [614]      0.0000             granulocyte monocyte progenitor cell
##   [615]      0.0000                        hematopoietic stem cell
##   [616]      0.0000         megakaryocyte-erythroid progenitor cell
##   [617]      0.0000 CD14-positive, CD16-negative classical monocyte
##   [618]      0.0000                 CD4-positive, alpha-beta T cell
##   -------
##   seqinfo: 3 sequences from an unspecified genome; no seqlengths
```

Tiling regions

We use the `deepblue_tiling_regions` command to generate a set of consecutive genomic regions of size 100,000 from chromosome 1 of the genome assembly GRCh38.

The command `deepblue_aggregate` summarizes the `query_id` regions using the column `VALUE` and the `cpg_islands` regions as boundaries.

```r
# Selecting the data from 2 experiments:
#    GC_T14_10.CPG_methylation_calls.bs_call.GRCh38.20160531.wig
# As we already know the experiments names, we keep all others fields empty.
# We are selecting all regions of chromosome 1
query_id = deepblue_select_experiments(
    experiment=c("GC_T14_10.CPG_methylation_calls.bs_call.GRCh38.20160531.wig"),
    chromosome="chr1")

#  Tiling regions of 100.000 base pairs
```

```r
tiling_id = deepblue_tiling_regions(size=100000,
    genome="GRCh38", chromosome="chr1")

# Aggregate
overlapped = deepblue_aggregate (data_id=query_id,
    ranges_id=tiling_id, column="VALUE")

# Retrieve the experiments data (The @NAME meta-column is used to include the
# experiment name and @BIOSOURCE for experiment's biosource
request_id = deepblue_get_regions(query_id=overlapped,
    output_format="CHROMOSOME,START,END,@AGG.MEAN,@AGG.SD")

regions = deepblue_download_request_data(request_id=request_id)
regions
```

```
## GRanges object with 2489 ranges and 2 metadata columns:
##          seqnames                 ranges strand | @AGG.MEAN   @AGG.SD
##             <Rle>              <IRanges>  <Rle> | <numeric> <numeric>
##      [1]     chr1       [     0, 100000]      * |    0.6677    0.3639
##      [2]     chr1       [100000, 200000]      * |    0.8358    0.2414
##      [3]     chr1       [200000, 300000]      * |    0.7714    0.2512
##      [4]     chr1       [300000, 400000]      * |    0.7595    0.2477
##      [5]     chr1       [400000, 500000]      * |    0.8512    0.1877
##      ...      ...                    ...    ... .       ...       ...
##   [2485]     chr1 [248400000, 248500000]      * |    0.8348     0.188
##   [2486]     chr1 [248500000, 248600000]      * |    0.8576    0.1561
##   [2487]     chr1 [248600000, 248700000]      * |    0.8664    0.1786
##   [2488]     chr1 [248700000, 248800000]      * |    0.8425    0.1846
##   [2489]     chr1 [248800000, 248900000]      * |    0.6572    0.4079
##   -------
##   seqinfo: 1 sequence from an unspecified genome; no seqlengths
```

Such data can now be plotted using any of the common R plotting mechanisms and packages. An example is shown here:

```r
library(ggplot2)
plot_data <- as.data.frame(regions)
plot_data[,grepl("X.", colnames(plot_data))] <-
    apply(plot_data[,grepl("X.", colnames(plot_data))], 2, as.numeric)
AGG.plot <- ggplot(plot_data, aes(start)) +
    geom_ribbon(aes(ymin = X.AGG.MEAN - (X.AGG.SD / 2),
        ymax = X.AGG.MEAN + (X.AGG.SD / 2)), fill = "grey70") +
    geom_line(aes(y = X.AGG.MEAN))
print(AGG.plot)
```

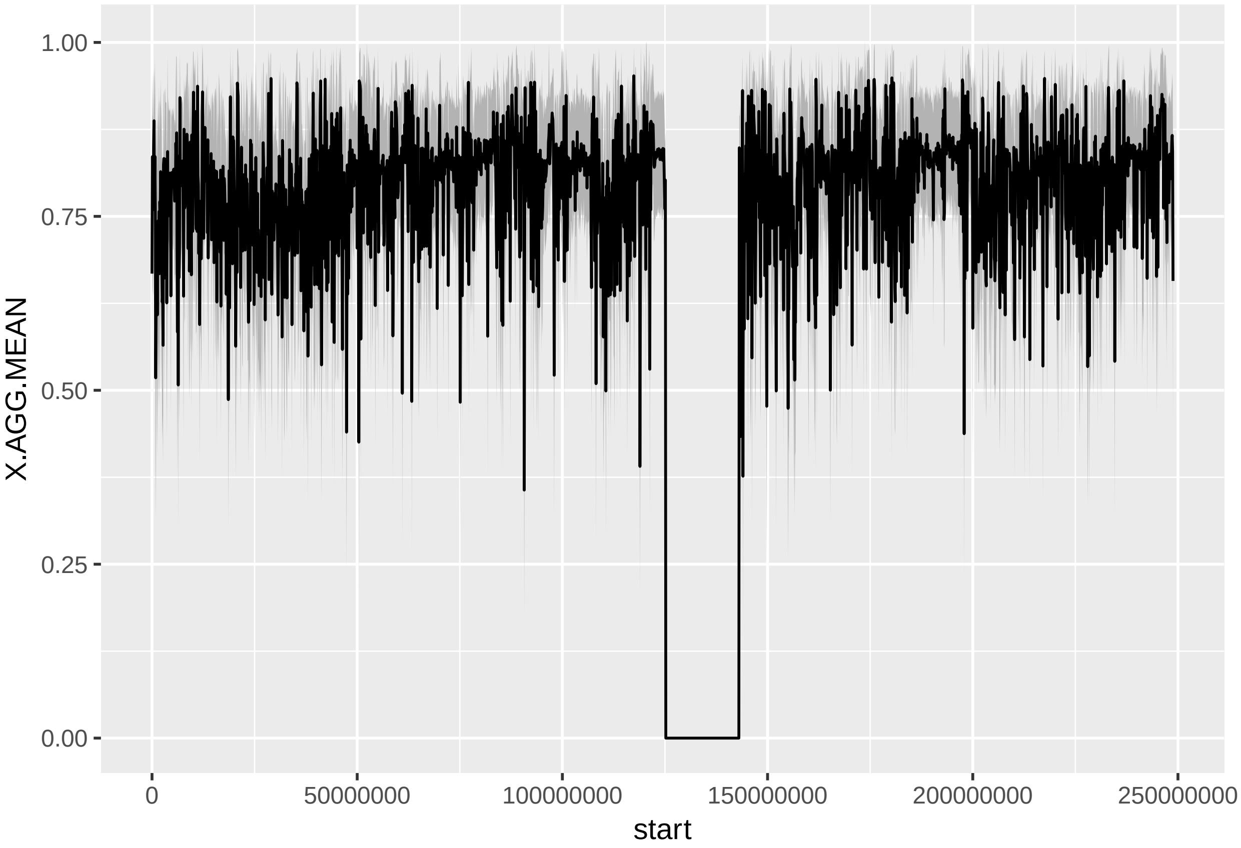

Flanking regions

We use the deepblue_select_genes command to generate a set of genes from the gene model GENCODE v19.

The deepblue_flank command derives flanking regions from existing regions. First, we derive regions that start 2500bp before the initially selected regions with a length of 2000bp. Next, we derive the regions that start 1500 base pairs after the initially selected regions with 500 base pairs length. For each region, we consider the DNA strand.

The deepblue_merge_queries command merges the region sets defined by two query IDs. Here, we merge the two flanking regions sets we created based on the initially selected genes.

```
# Select the RP11-34P13 gene locations from gencode v23
q_genes = deepblue_select_genes(
    genes = c("RNU6-1100P", "CICP7", "MRPL20", "ANKRD65",
            "HES2", "ACOT7", "HES3", "ICMT"),
    gene_model="gencode v19")

# Obtain the regions that starts 2500 bases pair before the regions start and
# have 2000 base pairs.
# The 4th argument inform that DeepBlue must consider the region strand
# (column STRAND) to calculate the new region
before_flank_id = deepblue_flank(query_id=q_genes,
     start=-2500, length=2000, use_strand=TRUE)

# Obtain the regions that starts 1500 bases pair after the
```

```r
# regions end and have 500 base pairs.
# The 4th argument inform that DeepBlue must consider the
# region strand (column STRAND) to calculate the new region
after_flank_id = deepblue_flank(query_id=q_genes,
    start=1500, length=500, use_strand=TRUE)

# Merge both flanking regions set and genes set
flank_merge_id = deepblue_merge_queries(
    query_a_id =before_flank_id, query_b_id=after_flank_id)
all_merge_id = deepblue_merge_queries(
    query_a_id=q_genes, query_b_id=flank_merge_id)

# Request the regions
request_id = deepblue_get_regions(query_id=all_merge_id,
    output_format="CHROMOSOME,START,END,STRAND,@LENGTH")

regions = deepblue_download_request_data(request_id=request_id)
regions
```

```
## GRanges object with 27 ranges and 1 metadata column:
##        seqnames               ranges strand |   @LENGTH
##           <Rle>            <IRanges>  <Rle> | <integer>
##    [1]     chr1     [155784, 156284]      - |       500
##    [2]     chr1     [157784, 157887]      - |       103
##    [3]     chr1     [160387, 162387]      - |      2000
##    [4]     chr1     [327431, 327931]      - |       500
##    [5]     chr1     [329431, 332236]      - |      2805
##    ...      ...                  ...    ... .       ...
##   [23]     chr1 [ 6472478,  6484730]      - |     12252
##   [24]     chr1 [ 6487230,  6489230]      - |      2000
##   [25]    chr21 [38364443, 38366443]      + |      2000
##   [26]    chr21 [38366943, 38367375]      + |       432
##   [27]    chr21 [38368875, 38369375]      + |       500
##   -------
##   seqinfo: 2 sequences from an unspecified genome; no seqlengths
```

Calculated columns

Here, we summarize DNA methylation levels for CpG islands of a specific experiment. Next, we remove those CpG islands for which no values were found using `@AGG.COUNT` > 0.

We use the `@CALCULATED` meta-column to transform the `@AGG.MEAN` value to log scale.

```r
# Select the RP11-34P13 gene locations from gencode v23

# Selecting the data from 2 experiments:
#    GC_T14_10.CPG_methylation_calls.bs_call.GRCh38.20160531.wig
# As we already know the experiments names, we keep all others fields empty.
# We are selecting all regions of chromosome 1
query_id = deepblue_select_experiments(
    experiment="GC_T14_10.CPG_methylation_calls.bs_call.GRCh38.20160531.wig",
    chromosome="chr1")

# Select the CpG Islands annotation from GRCh38
```

```r
cpg_islands = deepblue_select_annotations(
    annotation="CpG Islands", genome="GRCh38", chromosome="chr1")

# Aggregate
overlapped = deepblue_aggregate(
    data_id=query_id, ranges_id=cpg_islands, column="VALUE")

# Select the aggregated regions that aggregated at least one region from the
# selected experiments (@AGG.COUNT > 0)
filtered = deepblue_filter_regions(query_id=overlapped,
    field="@AGG.COUNT", operation=">", value="0", type="number")

# We remove all regions where the aggregation mean is zero.
filtered_zeros = deepblue_filter_regions(query_id=filtered,
    field="@AGG.MEAN", operation="!=", value="0.0", type="number")

# Retrieve the experiments data (The @NAME meta-column is used to include the
# experiment name and @BIOSOURCE for experiment's biosource
request_id = deepblue_get_regions(query_id=filtered_zeros,
    output_format=
    "CHROMOSOME,START,END,@CALCULATED(return math.log(value_of('@AGG.MEAN'))),@AGG.MEAN,@AGG.COUNT")

regions = deepblue_download_request_data(request_id=request_id)

# We have to perform a manual conversion because the
# package can't know the type for calculated columns
regions$`@CALCULATED(return math.log(value_of('@AGG.MEAN')))` =
    as.numeric(regions$`@CALCULATED(return math.log(value_of('@AGG.MEAN')))`)

head(regions, 5)
```

```
## GRanges object with 5 ranges and 3 metadata columns:
##       seqnames           ranges strand |
##          <Rle>        <IRanges>  <Rle> |
##   [1]     chr1 [ 28735,  29737]      * |
##   [2]     chr1 [135124, 135563]      * |
##   [3]     chr1 [368792, 370063]      * |
##   [4]     chr1 [381172, 382185]      * |
##   [5]     chr1 [491107, 491546]      * |
##       @CALCULATED(return math.log(value_of('@AGG.MEAN'))) @AGG.MEAN
##                                                <numeric> <numeric>
##   [1]                                          -7.600902    0.0005
##   [2]                                          -0.083708    0.9197
##   [3]                                                  0         1
##   [4]                                           -0.04343    0.9575
##   [5]                                          -0.046044     0.955
##       @AGG.COUNT
##        <integer>
##   [1]         64
##   [2]         30
##   [3]          2
##   [4]         12
##   [5]         21
##   -------
```

```
##   seqinfo: 1 sequence from an unspecified genome; no seqlengths
```

Any numerical values returned by DeepBlue can also be conveniently displayed using, for example, the DataTrack feature of the GViz Bioconductor package as shown here:

```
library(Gviz)
atrack <- AnnotationTrack(regions,
    name = "CpGs", group = regions$`@BIOSOURCE`, genome="hg38")
gtrack <- GenomeAxisTrack()
itrack <- IdeogramTrack(genome = "hg38", chromosome = "chr1")
dtrack <- DataTrack(regions,
    data="@AGG.MEAN", name = "Log of average methylation value")
plotTracks(list(itrack, atrack, dtrack, gtrack), type="histogram", fontsize=18,
           background.panel = "#FFFEDB", background.title = "darkblue")
```

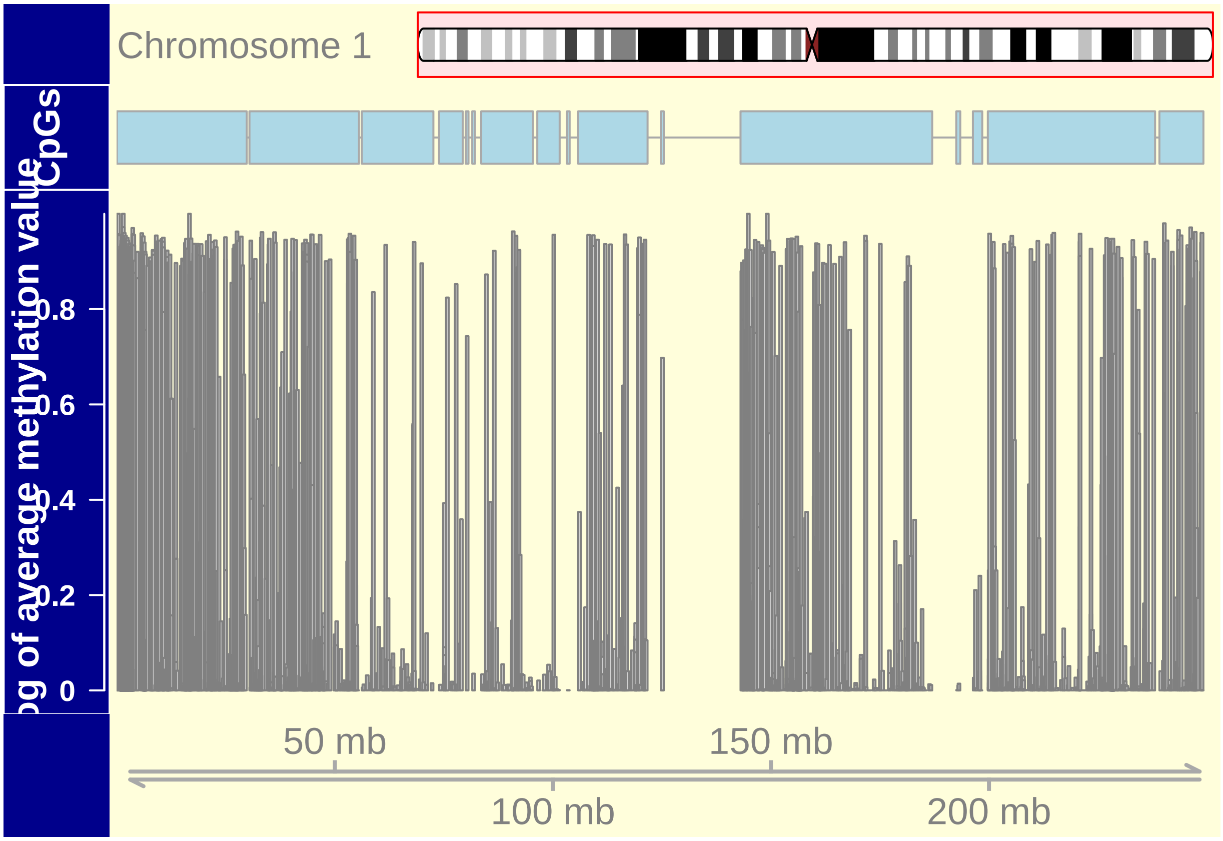

Score matrix

Here, we select a small number of experiments for which we want to build a score matrix based on the column `VALUE`.

We use CpG islands as aggregated regions boundaries.

The `deepblue_score_matrix` command expects a named list with the experiments names and columns that will be used for aggregation, the regions' boundaries, and the operation that will be performed (min, max, mean, var, sd, median, count).

The `deepblue_score_matrix` command is executed asynchronously. The command `download_request_data` will return a matrix in which the first three columns correspond to the chromosome, start position and

end position. The remaining columns will carry the names of the experiments and hold the corresponding aggregated values.

```
experiments =
    c("GC_T14_10.CPG_methylation_calls.bs_call.GRCh38.20160531.wig",
        "C003N351.CPG_methylation_calls.bs_call.GRCh38.20160531.wig",
        "C005VG51.CPG_methylation_calls.bs_call.GRCh38.20160531.wig",
        "S002R551.CPG_methylation_calls.bs_call.GRCh38.20160531.wig",
        "NBC_NC11_41.CPG_methylation_calls.bs_call.GRCh38.20160531.wig",
        "bmPCs-V156.CPG_methylation_calls.bs_call.GRCh38.20160531.wig",
        "S00BS451.CPG_methylation_calls.bs_call.GRCh38.20160531.wig",
        "S00D1DA1.CPG_methylation_calls.bs_call.GRCh38.20160531.wig",
        "S00D39A1.CPG_methylation_calls.bs_call.GRCh38.20160531.wig")

experiments_columns = list()
for (experiment_name in experiments) {
    experiments_columns[[experiment_name]] = "VALUE"
}

cpgs = deepblue_select_annotations(
    annotation_name="Cpg Islands",
    chromosome="chr22", start=0, end=18000000, genome="GRCh38")

request_id = deepblue_score_matrix(
    experiments_columns=experiments_columns,
    aggregation_function="mean", aggregation_regions_id=cpgs)

score_matrix = deepblue_download_request_data(request_id=request_id)
head(score_matrix, 5)
```

```
##    CHROMOSOME    START      END
## 1:      chr22 10525486 10527570
## 2:      chr22 10571557 10572827
## 3:      chr22 10698820 10699961
## 4:      chr22 10741251 10742442
## 5:      chr22 10961033 10961845
##    C003N351.CPG_methylation_calls.bs_call.GRCh38.20160531.wig
## 1:                                                          NA
## 2:                                                          NA
## 3:                                                          NA
## 4:                                                    0.846770
## 5:                                                    0.806492
##    C005VG51.CPG_methylation_calls.bs_call.GRCh38.20160531.wig
## 1:                                                          NA
## 2:                                                          NA
## 3:                                                          NA
## 4:                                                          NA
## 5:                                                    0.764457
##    GC_T14_10.CPG_methylation_calls.bs_call.GRCh38.20160531.wig
## 1:                                                           NA
## 2:                                                           NA
## 3:                                                           NA
## 4:                                                           NA
## 5:                                                           NA
##    NBC_NC11_41.CPG_methylation_calls.bs_call.GRCh38.20160531.wig
```

```
## 1:                                                                  NA
## 2:                                                                  NA
## 3:                                                                  NA
## 4:                                                                  NA
## 5:                                                            0.808091
##    S002R551.CPG_methylation_calls.bs_call.GRCh38.20160531.wig
## 1:                                                               NA
## 2:                                                               NA
## 3:                                                               NA
## 4:                                                               NA
## 5:                                                         0.629358
##    S00BS451.CPG_methylation_calls.bs_call.GRCh38.20160531.wig
## 1:                                                               NA
## 2:                                                               NA
## 3:                                                               NA
## 4:                                                         0.742429
## 5:                                                         0.717286
##    S00D1DA1.CPG_methylation_calls.bs_call.GRCh38.20160531.wig
## 1:                                                               NA
## 2:                                                               NA
## 3:                                                               NA
## 4:                                                               NA
## 5:                                                         0.805734
##    S00D39A1.CPG_methylation_calls.bs_call.GRCh38.20160531.wig
## 1:                                                               NA
## 2:                                                               NA
## 3:                                                               NA
## 4:                                                               NA
## 5:                                                               NA
##    bmPCs-V156.CPG_methylation_calls.bs_call.GRCh38.20160531.wig
## 1:                                                                 NA
## 2:                                                                 NA
## 3:                                                                 NA
## 4:                                                                 NA
## 5:                                                           0.775065
```

```
library(ggplot2)
score_matrix_plot = tidyr::gather(score_matrix,
    "experiment", "methylation", -CHROMOSOME, -START, -END)
score_matrix_plot$START <- as.factor(score_matrix_plot$START)
ggplot(score_matrix_plot, aes(x=START, y=experiment, fill=methylation)) +
    geom_tile() +
    theme(axis.text.x=element_text(angle=-90))
```

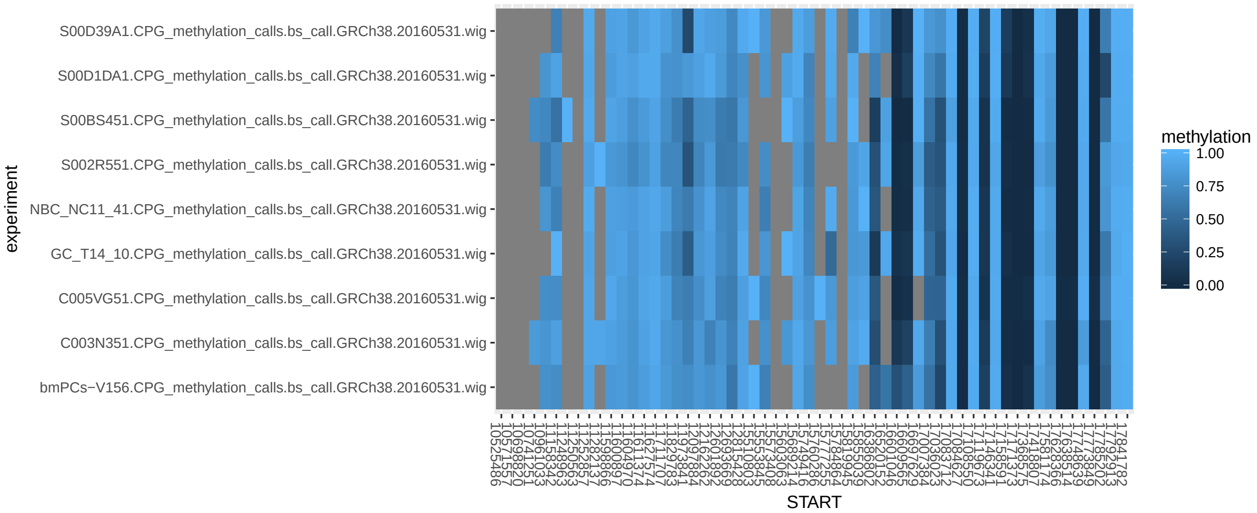

Data Export

DeepBlueR allows you to conveniently save results to disk. Any result can be saved as tab delimited file using `deepblue_export_tab`. For example, we can save the score matrix generated in the above example:

```
deepblue_export_tab(score_matrix, file.name = "my_score_matrix")
```

Results obtained with `deepblue_get_regions` are of type GenomicRanges and can be exported as tab delimited files preserving all columns or as BED files, where a specific column can optionally be selected to populate the 'score' column of the BED file. To demonstrate this, we use the result from the tiling regions example further above:

```
request_id = deepblue_get_regions(query_id=overlapped,
    output_format="CHROMOSOME,START,END,@AGG.MEAN,@AGG.SD")

regions = deepblue_download_request_data(request_id=request_id)
deepblue_export_bed(regions,
                    file.name = "my_tiling_regions",
                    score.field = "@AGG.MEAN")
```

Furthermore, metadata associated with any id can be stored locally using the `deepblue_export_meta_data` command. To this end, we first obtain the experiment id of the file we used in the tiling regions example.

```
exp_id <- deepblue_name_to_id(
    "GC_T14_10.CPG_methylation_calls.bs_call.GRCh38.20160531.wig",
    collection = "experiments")$id

deepblue_export_meta_data(exp_id, file.name = "GC_T14")
```

This command can also handle lists of ids, for instance:

```
deepblue_export_meta_data(list("e30035", "e30036"),
file.name = "test_export")
```

In some cases, users will perform a series of requests. We provide the command `deepblue_batch_export` to save these results and their associated metadata to disk in one go. This method will save each file as it becomes available, i.e. it will be saved once the request is successfully processed by DeepBlue:

```
experiments = deepblue_list_experiments(type="peaks", epigenetic_mark="H3K4me3",
    biosource=c("inflammatory macrophage", "macrophage"),
```

```r
    project="BLUEPRINT Epigenome")
experiment_names = deepblue_extract_names(experiments)

request_ids = foreach(experiment = experiment_names) %do%{
  query_id = deepblue_select_experiments(experiment_name = experiment,
                                        chromosome = "chr21")

  request_id = deepblue_get_regions(query_id =query_id,
    output_format = "CHROMOSOME,START,END")
}
request_data = deepblue_batch_export_results(request_ids,
                              target.directory = "BLUEPRINT macrophages chr21")
```

Options

DeepBlueR comes with default options that can be changed by the user. To list the current options use the following command:

```r
deepblue_options()
```

```
## $url
## [1] "http://deepblue.mpi-inf.mpg.de/xmlrpc"
##
## $user_key
## [1] "anonymous_key"
##
## $do_not_cache
## [1] FALSE
##
## $force_download
## [1] FALSE
##
## $debug
## [1] FALSE
```

- `url` This is the URL of the DeepBlue application server and should not be changed
- `user_key` This option can be replaced by the personal key of the user after successful registration at http://deepblue.mpi-inf.mpg.de. The key can be found by logging into the web application and clicking on the user name in the top left corner. Registered users have access to advanced features of DeepBlue, e.g. they can review previous requests.
- `do_not_cache` Allows users to switch off the caching functionality of DeepBlueR. See Caching.
- `force_download` If the users wishes to overwrite cached results for the following requests, this option can be switched on. See Caching.
- `debug` Switching on this option enables verbose output only useful for debugging.

Changing an option works as follows:

```r
deepblue_options(do_not_cache = TRUE)
```

Another example (replace 'my_user_key' with the actual key):

```r
deepblue_options(user_key = "my_user_key")
```

In case you wish to restore the default options simply call

```r
deepblue_reset_options()
```

Caching

DeepBlueR by default creates a file 'DeepBlueR.cache' in the current working directory. Downloaded results / regions are stored there and can be instantly retrieved, which is particularly useful for users with limited network bandwith. However, in case caching is not desired it can be switched off (see Options)

To check the status of the cache you can use the following command:

```r
deepblue_cache_status()
```

This will report the cache size and the number of requests currently stored. Alternatively, users can list the request ids for which results are available:

```r
deepblue_list_cached_requests()
```

Over time, the cache can quickly grow in size. It is possible to remove individual requests from the cache if the request id is known:

```r
deepblue_delete_request_from_cache("r123")
```

In most cases it will be simpler to simply delete the cache:

```r
deepblue_clear_cache()
```

Large-scale analysis of DNA methylation across 212 samples from the BLUEPRINT consortium

Aim

Here, we will show how DeepBlueR can be used to generate an overview heatmap of variable positions in more than 200 BLUEPRINT DNA methylation experiments. The amount of data considered here would normally be too huge to be processed on a local R installation. However, using DeepBlue and server-side processing of the data, we can facilitate this large-scale analysis easily.

Dependencies

In the first step, we load the DeepBlueR package, as well as packages for data retrieval, matrix operations and plotting.

```r
library(DeepBlueR)
library(gplots)
library(RColorBrewer)
library(matrixStats)
library(stringr)
```

Select experiments

Next, we list all available BLUEPRINT DNA methylation experiments. (412 files that match the required metadata were available during the edition of this vignette.)

```r
blueprint_DNA_meth <- deepblue_list_experiments(genome = "GRCh38",
                                   epigenetic_mark = "DNA Methylation",
                                   technique = "Bisulfite-Seq",
                                   project = "BLUEPRINT EPIGENOME")

blueprint_DNA_meth
```

```
##          id                                                  name
##   1: e93372   S00B2JA1.CPG_methylation_calls.bs_cov.GRCh38.20160531.wig
##   2: e93367  S00B2JA1.CPG_methylation_calls.bs_call.GRCh38.20160531.wig
##   3: e93356   C003VO56.CPG_methylation_calls.bs_cov.GRCh38.20160531.wig
##   4: e93353  C003VO56.CPG_methylation_calls.bs_call.GRCh38.20160531.wig
##   5: e93346 C0010KA2bs.CPG_methylation_calls.bs_cov.GRCh38.20160531.wig
##  ---
## 408: e95479   S016KWU1.CPG_methylation_calls.bs_cov.GRCh38.20160531.wig
## 409: e95527  S00D39A1.CPG_methylation_calls.bs_call.GRCh38.20160531.wig
## 410: e95528   S00D39A1.CPG_methylation_calls.bs_cov.GRCh38.20160531.wig
## 411: e95550  S013SSA1.CPG_methylation_calls.bs_call.GRCh38.20160531.wig
## 412: e95551   S013SSA1.CPG_methylation_calls.bs_cov.GRCh38.20160531.wig
```

We are only interested in a subset of those files and filter for call files (opposed to coverage files).

```r
blueprint_DNA_meth <- blueprint_DNA_meth[grep("bs_call",
    deepblue_extract_names(blueprint_DNA_meth)),]

blueprint_DNA_meth
```

```
##          id                                                   name
##   1: e93367   S00B2JA1.CPG_methylation_calls.bs_call.GRCh38.20160531.wig
##   2: e93353   C003VO56.CPG_methylation_calls.bs_call.GRCh38.20160531.wig
##   3: e93342   C003VO55.CPG_methylation_calls.bs_call.GRCh38.20160531.wig
##   4: e93341 C0010KA2bs.CPG_methylation_calls.bs_call.GRCh38.20160531.wig
##   5: e93332       P581.CPG_methylation_calls.bs_call.GRCh38.20160531.wig
##  ---
## 202: e95397   S00D2BA1.CPG_methylation_calls.bs_call.GRCh38.20160531.wig
## 203: e95436   S00Y05A1.CPG_methylation_calls.bs_call.GRCh38.20160531.wig
## 204: e95478   S016KWU1.CPG_methylation_calls.bs_call.GRCh38.20160531.wig
## 205: e95527   S00D39A1.CPG_methylation_calls.bs_call.GRCh38.20160531.wig
## 206: e95550   S013SSA1.CPG_methylation_calls.bs_call.GRCh38.20160531.wig
```

Select experiment column

Each of these files has a column, named `VALUE`, that holds the DNA methylation beta values. There are two possibilities to select this column across several files.

First, we assume that the column in question has a different name in each file. We thus have to create a list that holds the column name for each of them. Such a list can be generated using standard R commands:

```r
exp_columns <- list(nrow(blueprint_DNA_meth))

for(i in 1:nrow(blueprint_DNA_meth)){
    exp_columns[[i]] <- "VALUE"
}
names(exp_columns) <- deepblue_extract_names(blueprint_DNA_meth)
```

In most cases, the same column name will apply for each file. We thus implemented a short hand function for generating the above list with a single column name for all files.

```
exp_columns <- deepblue_select_column(blueprint_DNA_meth, "VALUE")
```

Filter for genomic regions of interest using annotations

In the next operation, we consider that not all methylation sites will be informative for clustering the data. We thus filter for those regions that are part of the BLUEPRINT regulatory build, a modified version of the ENSEMBL regulatory build that contains promoters, promoter flanking regions, enhancers, CTCF binding sites, transcription factor binding sites and open chromatin regions. As we can see, DeepBlueR returns a query id, which we store for later use.

```
blueprint_regulatory_regions <- deepblue_select_annotations(
    annotation_name = "Blueprint Ensembl Regulatory Build",
    genome = "GRCh38")

blueprint_regulatory_regions
```

```
## [1] "q917484"
```

DeepBlue has several annotations that can be used to filter for informative sites. We could, for example, also filter for CpG islands.

```
deepblue_select_annotations(annotation_name = "Cpg Islands",
                            genome = "GRCh38")
```

A list of all annotations currently available for a genome is given by the following command.

```
deepblue_list_annotations(genome = "GRCh38")
```

```
##       id                                            name
##  1:  a49                          Chromosomes size for GRCh38
##  2: a132                                           promoters
##  3: a161    Pattern TATAAA (non-overlap) in the genome GRCh38
##  4: a164                                         Cpg Islands
##  5: a239      Pattern TATA (non-overlap) in the genome GRCh38
##  6: a240        Pattern GC (non-overlap) in the genome GRCh38
##  7: a241        Pattern CG (non-overlap) in the genome GRCh38
##  8: a242 Pattern (TATA|CG) (non-overlap) in the genome GRCh38
##  9: a243         Pattern G (non-overlap) in the genome GRCh38
## 10: a244         Pattern C (non-overlap) in the genome GRCh38
## 11: a245       Pattern C|G (non-overlap) in the genome GRCh38
## 12: a246         Pattern A (non-overlap) in the genome GRCh38
## 13: a249                  Blueprint Ensembl Regulatory Build
```

New annotations may be included upon users request.

In case we want to include the entire genome in an aggregated version DeepBlue supports the concept of tiling regions. In this process, the genomic range of interest will be binned into tiles of a given size (here 5kb).

```
tiling_regions <- deepblue_tiling_regions(size=5000,
                                          genome="GRCh38")
```

Generate a score matrix

In the above step we have defined a set of regions of interest that we want to interrogate in R to, for example, cluster samples. To this end, DeepBlue can build a score matrix, in which the selected genomic regions are aggregated on the server to reduce the complexity and size of the data. We request such a score matrix in which regulatory regions are aggregated by the mean as follows. Note that we use the variables 'exp_columns' and 'blueprint_regulatory_regions' that we have defined above.

```
request_id <- deepblue_score_matrix(
    experiments_columns = exp_columns,
    aggregation_function = "mean",
    aggregation_regions_id = blueprint_regulatory_regions)

request_id
```

```
## [1] "r789199"
```

After triggering this function, DeepBlue queues our task and will execute it when resources become available. We also observe that DeepBlue returns a request id, which we can use to query the status of the operation.

```
deepblue_info(request_id)$state
```

```
## [1] "done"
```

When the operation is finished, we can download the score matrix and store it in a local variable. For DeepBlueR, we implemented several strategies to improve the performance of data retrieval. For instance, we modified the existing XML-RPC package to be more efficient in the context of DeepBlue when it comes to parsing nested XML data. Moreover, we retrieve tabular data directly in a tab separated file format, which can be processed much faster in R. Finally, we also compress data on the server side to reduce download time. Here, we only show the first five columns out of 215.

```
score_matrix <- deepblue_download_request_data(request_id = request_id)
```

```
##
Read 0.0% of 528248 rows
Read 7.6% of 528248 rows
Read 15.1% of 528248 rows
Read 22.7% of 528248 rows
Read 30.3% of 528248 rows
Read 37.9% of 528248 rows
Read 45.4% of 528248 rows
Read 53.0% of 528248 rows
Read 60.6% of 528248 rows
Read 68.1% of 528248 rows
Read 75.7% of 528248 rows
Read 83.3% of 528248 rows
Read 90.9% of 528248 rows
Read 98.4% of 528248 rows
Read 528248 rows and 209 (of 209) columns from 0.735 GB file in 00:00:21
```

```
score_matrix[,1:5, with=FALSE]
```

```
##        CHROMOSOME     START       END
##     1:       chr1     16047     30847
##     2:       chr1     19599     20609
##     3:       chr1     20892     21536
##     4:       chr1     24567     24911
##     5:       chr1     25804     26294
```

```
##     ---
## 528244:       chrX 156024218 156024915
## 528245:       chrX 156024974 156025400
## 528246:       chrX 156026242 156028242
## 528247:       chrX 156026442 156028042
## 528248:       chrY      6144      6409
##         C000S5A1bs.CPG_methylation_calls.bs_call.GRCh38.20160531.wig
##      1:                                                     0.581450
##      2:                                                     0.946625
##      3:                                                     0.750000
##      4:                                                     0.922500
##      5:                                                     1.000000
##     ---
## 528244:                                                     0.873444
## 528245:                                                     0.622000
## 528246:                                                     0.844286
## 528247:                                                     0.914824
## 528248:                                                           NA
##         C000S5A2bs.CPG_methylation_calls.bs_call.GRCh38.20160531.wig
##      1:                                                     0.681173
##      2:                                                     0.936800
##      3:                                                           NA
##      4:                                                           NA
##      5:                                                     1.000000
##     ---
## 528244:                                                     0.830529
## 528245:                                                     0.596308
## 528246:                                                     0.700850
## 528247:                                                     0.761688
## 528248:                                                           NA
```

The download is 212.8 MB in size. The size of the data we handled on DeepBlue to extract this information is roughly 212 x ~450 MB ~= 95 GB and thus more than can be handled in R on most desktop computers. We next show how this score matrix can be used to plot a heatmap where samples are clustered by the Pearson correlation coefficient, revealing that samples originating from the same cell type are more similar in DNA methylation.

Generating a heatmap

Metadata and colors

In preparation of the heatmap plot, we need to generate an RColorBrewer palette. This allows us to create a color palette for more than 9 colors.

```
getPalette <- colorRampPalette(brewer.pal(9, "Set1"))
```

For each experiment, we collect metadata.

```
experiments_info <- deepblue_info(deepblue_extract_ids(blueprint_DNA_meth))
```

All metadata is parsed to a nested R list. We refer to the DeepBlue paper for a description of available metadata. Here, we show the metadata associated with just one of the samples.

```
head(experiments_info[[1]], 10)
```

```
## $type
```

```
## [1] "experiment"
##
## $`_id`
## [1] "e93367"
##
## $data_type
## [1] "signal"
##
## $description
## [1] ""
##
## $epigenetic_mark
## [1] "dna methylation"
##
## $format
## [1] "CHROMOSOME,START,END,VALUE"
##
## $genome
## [1] "GRCh38"
##
## $name
## [1] "S00B2JA1.CPG_methylation_calls.bs_call.GRCh38.20160531.wig"
##
## $project
## [1] "BLUEPRINT Epigenome"
##
## $sample_id
## [1] "s10726"
```

For this analysis, we are only interested in the biosource name, i.e. the cell type. We can retrieve this information using standard R syntax. Note that we show only the first 6 entries here.

```
biosource <- unlist(lapply(experiments_info, function(x){ x$sample_info$biosource_name}))
head(biosource)
```

```
## [1] "venous blood"
## [2] "central memory CD8-positive, alpha-beta T cell"
## [3] "CD8-positive, alpha-beta T cell"
## [4] "CD14-positive, CD16-negative classical monocyte"
## [5] "CD4-positive, alpha-beta T cell"
## [6] "germinal center B cell"
```

To save some space on the plot, we replace positive with + and negative with -.

```
biosource <- str_replace_all(biosource, "-positive", "+")
biosource <- str_replace_all(biosource, "-negative", "-")
```

For the same reason, we remove the words ‘terminally differentiated’ from one of the cell types.

```
biosource <- str_replace(biosource, ", terminally differentiated", "")
```

Using above color palette, we can now assign a unique color to each cell type.

```
color_map <- data.frame(biosource = unique(biosource),
                        color = getPalette(length(unique(biosource))))

head(color_map)
```

```
##                                 biosource   color
## 1                             venous blood #E41A1C
## 2 central memory CD8+, alpha-beta T cell #C52B37
## 3                 CD8+, alpha-beta T cell #A63D53
## 4        CD14+, CD16- classical monocyte #874F6F
## 5                 CD4+, alpha-beta T cell #68618A
## 6                  germinal center B cell #4A72A6
```

Using above table, we can now assign the colors to each experiment according to its cell type / biosource.

```
exp_names <- unlist(lapply(experiments_info, function(x){ x$name}))

biosource_colors <- data.frame(name = exp_names, biosource = biosource)
biosource_colors <- dplyr::left_join(biosource_colors, color_map, by = "biosource")
head(biosource_colors)
```

```
##                                                              name
## 1   S00B2JA1.CPG_methylation_calls.bs_call.GRCh38.20160531.wig
## 2   C003VO56.CPG_methylation_calls.bs_call.GRCh38.20160531.wig
## 3   C003VO55.CPG_methylation_calls.bs_call.GRCh38.20160531.wig
## 4 C0010KA2bs.CPG_methylation_calls.bs_call.GRCh38.20160531.wig
## 5       P581.CPG_methylation_calls.bs_call.GRCh38.20160531.wig
## 6       G201.CPG_methylation_calls.bs_call.GRCh38.20160531.wig
##                                 biosource   color
## 1                             venous blood #E41A1C
## 2 central memory CD8+, alpha-beta T cell #C52B37
## 3                 CD8+, alpha-beta T cell #A63D53
## 4        CD14+, CD16- classical monocyte #874F6F
## 5                 CD4+, alpha-beta T cell #68618A
## 6                  germinal center B cell #4A72A6
```

Finally, we transform this data frame into a vector that is compatible with the heatmap function.

```
color_vector <- as.character(biosource_colors$color)
names(color_vector) <-  biosource_colors$biosource
head(color_vector)
```

```
##                           venous blood
##                              "#E41A1C"
## central memory CD8+, alpha-beta T cell
##                              "#C52B37"
##                CD8+, alpha-beta T cell
##                              "#A63D53"
##       CD14+, CD16- classical monocyte
##                              "#874F6F"
##                CD4+, alpha-beta T cell
##                              "#68618A"
##                 germinal center B cell
##                              "#4A72A6"
```

Processing the input data

We remove the first three columns (CHROMOSOME, START, END) and convert the data frame to a numeric matrix.

```r
filtered_score_matrix <- as.matrix(score_matrix[,-c(1:3), with=FALSE])
head(filtered_score_matrix[,1:3])
```

```
##      C000S5A1bs.CPG_methylation_calls.bs_call.GRCh38.20160531.wig
## [1,]                                                     0.5814500
## [2,]                                                     0.9466250
## [3,]                                                     0.7500000
## [4,]                                                     0.9225000
## [5,]                                                     1.0000000
## [6,]                                                     0.0903333
##      C000S5A2bs.CPG_methylation_calls.bs_call.GRCh38.20160531.wig
## [1,]                                                      0.681173
## [2,]                                                      0.936800
## [3,]                                                            NA
## [4,]                                                            NA
## [5,]                                                      1.000000
## [6,]                                                      0.191537
##      C0010KA1bs.CPG_methylation_calls.bs_call.GRCh38.20160531.wig
## [1,]                                                     0.5392500
## [2,]                                                     0.9498670
## [3,]                                                            NA
## [4,]                                                     0.9666670
## [5,]                                                     0.6845000
## [6,]                                                     0.0970745
```

Next, we compute the variance of each row and retain only genomic regions with variance > 0.05 for plotting. Plotting all regions would consume too much memory and more importantly, regions that do not show variance also do not allow us to spot differences between cell types in the heatmap.

```r
message("regions before: ", nrow(filtered_score_matrix))
filtered_score_matrix_rowVars <- rowVars(filtered_score_matrix, na.rm = TRUE)
filtered_score_matrix <- filtered_score_matrix[which(filtered_score_matrix_rowVars > 0.05),]
message("regions after: ", nrow(filtered_score_matrix))
```

To be able to cluster samples, we remove regions that have missing values in at least one of the experiments.

```r
message("regions before: ", nrow(filtered_score_matrix))
filtered_score_matrix <- filtered_score_matrix[which(complete.cases(filtered_score_matrix)),]
message("regions after: ", nrow(filtered_score_matrix))
```

IMPORTANT: The order of columns in the score matrix is not the same as in the exp_columns list used in the request. We thus have to order the matrix by the experiment names in the color map. This is crucial to make sure we assign the correct cell type to each sample!

```r
filtered_score_matrix <- filtered_score_matrix[,exp_names]
```

Plotting

We plot a heatmap in which the variable regions are shown across all samples. On top of the columns, we create a dendrogram based on Pearson correlation More precisely, we convert the Pearson correlation, a similarity measure, to a distance, such that it can be used with hierarchical clustering.

```r
 heatmap.2(filtered_score_matrix,labRow = NA, labCol = NA,
          trace = "none", ColSideColors = color_vector,
          hclust=function(x) hclust(x,method="complete"),
          distfun=function(x) as.dist(1-cor(t(x), method = "pearson")),
```

```
        Rowv = TRUE, dendrogram = "column",
        key.xlab = "beta value", denscol = "black", keysize = 1.5,
        key.title = NA)
```

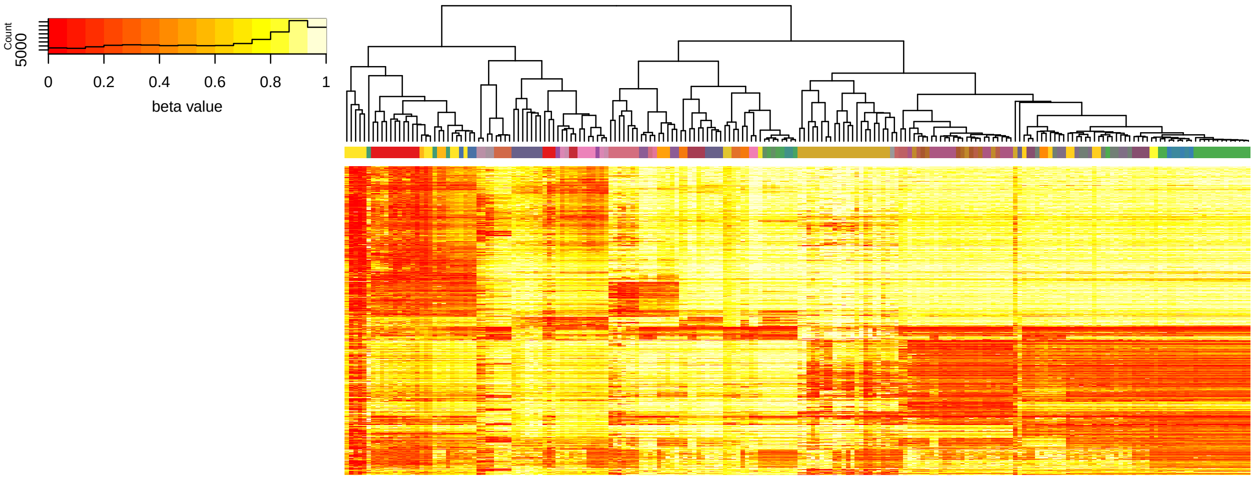


```
plot.new()

legend(x = 0, y = 1,
     legend = color_map$biosource,
     col = as.character(color_map$color),
     text.width = 0.6,
     lty= 1,
     lwd = 6,
     cex = 0.7,
     y.intersp = 0.7,
     x.intersp = 0.7,
     inset=c(-0.21,-0.11))
```

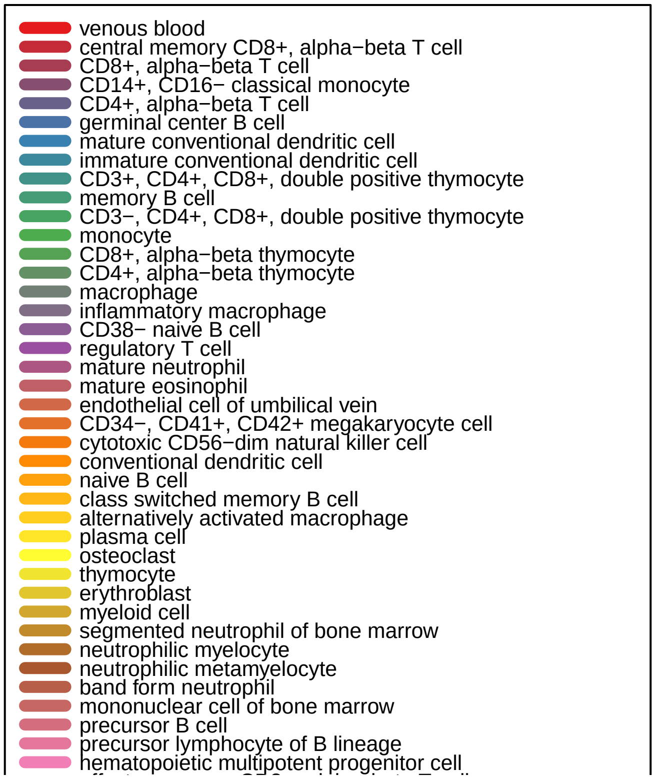

Further reading material

To obtain a general overview of DeepBlue, we recommend starting with the DeepBlue publication and a list of all DeepBlue commands is available in its API page.

You can have a look at the other use cases included in the R package and list them with

```
demo(package = "DeepBlueR")
```

Individual use cases can be triggered with

```
demo("use_case1", package = "DeepBlueR")
```

Note that the example presented here corresponds to use case 4 in the R package.

Final remarks

We encourage you to try to reproduce Python examples in R and to read the DeepBlue manual.

Moreover, we want to highlight the possibility to browse and access existing data in DeepBlue conveniently in the web interface. The web interface also allows you to select experiments in a grid like view.

Should you encounter any problems with DeepBlueR, we kindly ask you to create an issue in the BioConductor DeepBlueR support page.

The R code in the DeepBlueR package is under the GPLv3 license and we welcome contributions of other developers. Finally, we would like to thank the Bioconductor team for its support in making DeepBlueR available to a wide audience of users.
